# Adverse Reaction of Sodium Hypochlorite during Endodontic Treatment of Primary Teeth

**DOI:** 10.5005/jp-journals-10005-1304

**Published:** 2015-08-11

**Authors:** Vishwas Bhausaheb Chaugule, Amey Manohar Panse, Pritesh Namdeo Gawali

**Affiliations:** Professor and Head, Department of Pedodontics, Sinhgad Dental College, Pune Maharashtra, India; Lecturer, Department of Pedodontics, Sinhgad Dental College, Pune Maharashtra, India; Postgraduate Student, Department of Pedodontics, Sinhgad Dental College, Pune Maharashtra, India

**Keywords:** Complication, Root canal irrigation, Sodium hypochlorite.

## Abstract

Sodium hypochlorite (NaOCl) is the most common and effective intracanal medicament used in root canal treatments, because of its low-cost and a very effective antimicrobial activity against microbiota of infected root canals. Sodium hypochlorite is an effective intracanal irrigant and is used in concentrations ranging from 0.5 to 5.25%. At these concentrations, it is highly hypertonic and strongly alkaline with pH 11 to 13. Despite its safe properties, serious complications can result from inadvertent use due to its cytotoxic features. Most of the complications are the result of accidental extrusion of the solution from the apical foramen or accessory canals or perforations into the periapical area. Although it is an effective solution for disinfection of root canal system, fewer incidence of complications are reported, especially in primary teeth. Present article highlights one of such cases of NaOCl accident and its successful management in a 4-year-old child.

**How to cite this article:** Chaugule VB, Panse AM, Gawali PN. Adverse Reaction of Sodium Hypochlorite during Endo-dontic Treatment of Primary Teeth. Int J Clin Pediatr Dent 2015;8(2):153-156.

## INTRODUCTION

The motive behind root canal cleaning and shaping is the elimination of tissue remnants, bacteria, and toxins from the root canal system. Mechanical procedures alone are insufficient for total canal cleaning. Residual pulpal tissue, bacteria, and dentin debris may persist in the irregularities of canal systems. Therefore, irrigating solutions should support and complement endodontic preparation. These irrigants should flush out dentin debris, dissolve organic tissue, disinfect the canal system, and provide lubrication during instrumentation, without irritating the surrounding tissues. Some of the irrigants currently used include hydrogen peroxide, physiologic saline, water, sodium hypochlorite (NaOCl), chlorhexidine, and electrochemically activated water.^1^

Sodium hypochlorite was first recommended as an antiseptic solution by Henry Dakin in 1915 during 1st world war. Dakin’s solution, i.e. 0.5% of NaOCl buffered with NaHCO_3_ was used for dressing of wounds.^2^ In 1920, Crane described its use for root canal debridement and sterilization.^3^ Since then it has gained popularity as an effective intracanal irrigant. A variety of NaOCl concentrations ranging from 0.5 to 5.25% have been advocated.^1^ At these concentrations, it is highly hypertonic and strongly alkaline with pH 11 to 13. It has strong proteolytic and oxidative properties. It can dissolve both necrotic and vital pulp tissue and can kill broad range of pathogens like Gram +ve, Gram -ve bacteria, fungi and viruses. Generally, the solution is instilled into the canals during and after mechanical preparation with a disposable plastic syringe with a fine needle attached.^4^ Several complications have been described in the literature during root canal irrigation with NaOCl inadvertently penetrating through the apical foramen or allergic reactions to the irrigant.^4,5^ Any irrigant, regardless of toxicity, has the potential to cause problems if extruded into periradicular tissues. The inadvertent penetration of the irrigant can occur in the event of wide apical foramina or the destruction of apical constriction during canal preparation or due to external resorption. Additionally, extreme pressure during irrigation or binding of the irrigation needle tip with no release for the irrigant can cause apical extrusion.^6^

A case of inadvertent pushing of NaOCl beyond the root apex to the periapical tissues during the root canal treatment in a 4-year-old girl is presented below:

## CASE REPORT

**Figs 1A and B:**
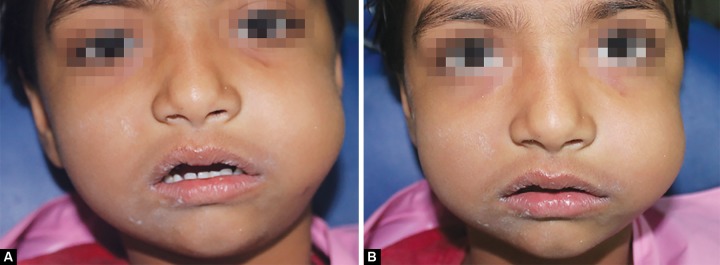
Swelling extending from the left infraorbital margin to angle of mandible

A 4-year-old girl reported to the Department of Pedodontics and Preventive Dentistry, Sinhgad Dental College and Hospital with chief complaint of a carious lesion in the upper left posterior region. On clinical examination, a deep occlusal carious lesion approaching the pulp was seen in upper left 1st and 2nd primary molars. No periapical pathology was evident. Radiographic interpretation suggested the need of pulpectomy for both primary molars. Pulpectomy procedure was initiated after administration of the local anesthesia. Access cavity was prepared and the working length was determined. Intermittent canal irrigation with 3% NaOCl was carried out whilst the biomechanical preparation of root canals was on. During instrumentation, due to profuse bleeding, copious irrigation with hypochlorite had to be carried out. While irrigating a sudden spontaneous extraoral swelling was observed on the left side of the face which increased gradually extending from the left infraorbital margin to angle of mandible and the patient experienced severe pain (Figs 1A and B). On palpation, the swelling was soft and diffuse. Crepitus was felt on palpation of periorbital swelling and mouth opening was not at all restricted.

Immediately, NaOCl accident was suspected. Canal was copiously irrigated with normal saline to dilute the effect of NaOCl concentration. An open dressing was given and cold compression with ice packs was given to control swelling and pain. The parents were informed about the cause and nature of the incident and reassured that a normal appearance will be regained within a short time. The patient was prescribed with antibiotic Cefadur 125 mg (1st generation cephalosporin) twice daily for 5 days, oral analgesic tablet Ibuclin Jr (Paracetamol 125 mg + Ibuprofen 100 mg) t.i.d for 3 days, syrup Atarax (1st Generation Antihistamine) 5 ml thrice daily and Tablet Deflazacort (Corticosteroid) 6 mg b.i.d. The parents were given appropriate home care instructions about feeding the child with soft diet and restricting oral move moments to reduce stress. The parents were advised to replace the cold compressions by hot ones after 24 hours to stimulate local systemic circulation. Upon constant communication with the parents, it was revealed that the patient’s both swelling and pain were at the bay level and the recovery appeared to be uneventful.

**Fig. 2:**
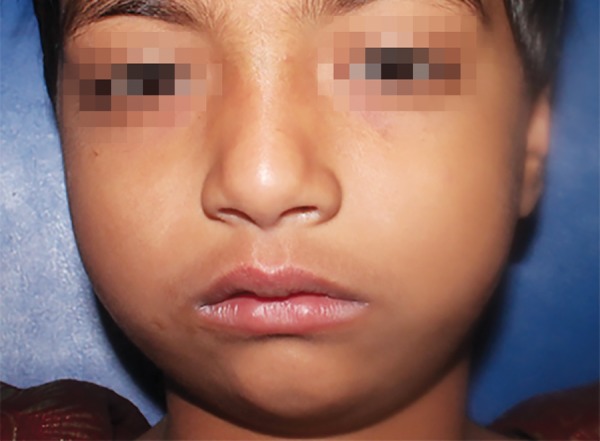
Reduced swelling on the 3rd day

**Fig. 3:**
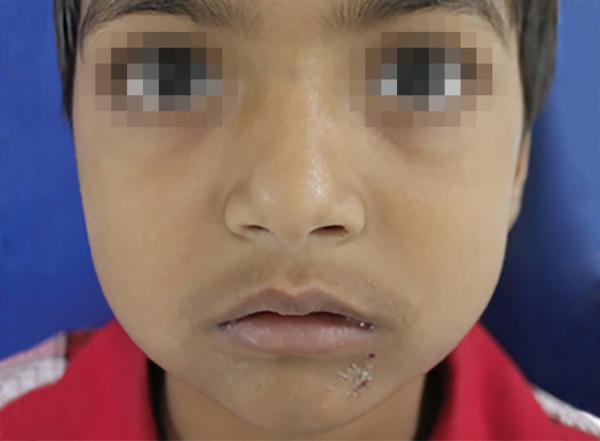
Clinical picture of the patient after 7 days showing normal tissue contour

On the 3rd day, the swelling reduced gradually, however a mild swelling was still evident (Fig. 2). Patient was regularly monitored at the subsequent recall appointments. Swelling completely disappeared on the 7th day and the patient regained her previous facial appearance (Fig. 3). Endodontic treatment of the affected teeth was re-initiated thereafter and completed.

## DISCUSSION

Due to the cytotoxic nature of NaOCl, occasional complications have been cited in the literature. When it comes into contact with tissue, it causes hemolysis and ulceration, inhibits neutrophil migration, and damages endothelial and fibroblast cells.^7,8^ Overshooting of the working length, lateral perforation, and wedging of the irrigating needle are the most common etiological factors associated with adverse NaOCl reactions.^9^ The majority of reported cases of sodium hypochlorite accident occurred as a result of forceful extrusion of NaOCl into the dental root, either by placing the irrigating syringe too far apically or using too high pressure, which pushes the irrigating solution into the periapical tissues.^10^ According to previous reports, extrusion of NaOCl solution into periradicular tissues during root canal treatment occurs more readily when there is a perforation or wide apex in the treated tooth combined with uncontrolled irrigation force. Sodium hypochlorite causes vascular permeability in blood vessels, probably as a result of damage to the vessels as well as the release of chemical mediators, such as histamine, from involved tissue. This behavior of NaOCl with tissue results into immediate swelling and profuse bleeding through the root canal when NaOCl is not used properly.^11^ Damage to permanent tooth follicles, peripheral tissue, and oral mucosa have been reported during negligent use of NaOCl use in pediatric endo-dontics.^12^ According to Hulsmann criteria, the diagnostic features of for a NaOCl accident include: (1) acute pain, swelling and redness; (2) bruising; (3) progressive swelling involving the infraorbital area or angle of mouth; (4) profuse hemorrhage often manifesting intraorally from the orifice of the tooth; (5) numbness or weakness of the facial nerve; and (6) secondary infection, sinusitis and cellulitis. In this case, distinguishing features included immediate diffuse infraorbital swelling, profuse hemorrhage and acute pain. Studies on the incidence of NaOCl accident showed that there was higher risk of NaOCl accident in endodontic treatment involving maxillary posterior teeth because of the thin cortical bone cover of the buccal roots.^13^ If an incident such as described in this case occurs, the dentist should remain calm. No specific treatment can reverse the damage from NaOCl. The mainstay of treatment is supportive including control of swelling, pain relief and prevention of secondary infec-tion.^10,14,15^ Analgesics should be given in order to relieve the post incident pain. A course of antibiotics should be prescribed, as there is potential for secondary or spread of infection. Local anesthesia in the presence of diffuse swelling should be avoided to prevent spreading of existing infection.^16^ Extraoral cold compresses should be used for the first 6 hours in order to minimize swelling. Subsequent to this initial period, heat packs should be used after 24 hours (15 minute interval) to improve the circulation to the area. According to Kleir et al, most of the patients recovered completely within one week, which is in accordance with the present case report.

The following steps can help clinicians avoid NaOCl accidents:

- Adequate access preparation.
- Good working length control.
- Irrigation needle should be placed 1 to 3 mm short of working length.
- Passive needle tip placement within the canal without getting bound to the walls thus permitting free movements of the needle.
- Irrigant to be expressed into the root canal gently with a low, constant pressure, withdrawing the needle slightly from the binding point.
- Use Luer Lock needles with side-port delivery that are specifically designed for endodontic purposes.

## CONCLUSION

This and similar kinds of report lead us to conclude that such incidences, though rare, are not uncommon. So to avoid discomfort and further complications, it would be prudent to take up precautions cited earlier. Delicate, careful handling of the already inflamed tissue is the key factor in keeping the patient comfortable and happy.
